# The Cohort of Indonesian Preterm Infants for Long-term Outcomes (CIPTO) study: a protocol

**DOI:** 10.1186/s12887-023-04263-z

**Published:** 2023-10-19

**Authors:** Rinawati Rohsiswatmo, Hardya Gustada Hikmahrachim, Muhamad Azharry Rully Sjahrulla, Putri Maharani Tristanita Marsubrin, Risma Kerina Kaban, Rosalina Dewi Roeslani, Adhi Teguh Perma Iskandar, Distyayu Sukarja, Ahmad Kautsar, Ivo Urwah, Hartono Gunardi, Yoga Devaera

**Affiliations:** 1 Division of Perinatology, Department of Child Health, Faculty of Medicine Universitas Indonesia, Cipto Mangunkusumo General Hospital, Jakarta, Indonesia; 2 Division of Growth and Development Social Pediatrics, Department of Child Health, Faculty of Medicine Universitas Indonesia, Cipto Mangunkusumo General Hospital, Jakarta, Indonesia; 3 Division of Nutrition and Metabolic Disease, Department of Child Health, Faculty of Medicine Universitas Indonesia, Cipto Mangunkusumo General Hospital, Jakarta, Indonesia

**Keywords:** Growth, Indonesia, Long-term outcomes, Neurodevelopment, Preterm infants, Prospective cohort

## Abstract

**Background:**

Indonesia has high numbers of preterm birth, i.e., around 600,000 preterm births annually. It imposes a significant burden on the Indonesia’s healthcare system. Indonesia therefore requires its own evidence-based reference to manage premature neonates and ex-preterm infants who subsequently survived. No long-term study on preterm infants in Indonesia has been conducted, therefore we aim to evaluate growth and development on ex-preterm infants until the pre-pubertal stage.

**Methods:**

We at the Cipto Mangunkusumo General Hospital (CMGH) designed a prospective cohort study of preterm infants, i.e., the Cohort of Indonesian Preterm Infants for Long-term Outcomes (CIPTO) study. At least 500 subjects will be recruited with an estimation of two-year recruitment (i.e., the recruitment phase will be completed before 2024). The CIPTO study will observe long-term outcomes of ex-preterm infants, primarily on growth and developmental milestones until 8 years old. Aims of this study are to determine the ex-preterm outcomes and to generate an evidence-based reference of preterm care for ensuring optimum outcomes. The pre-specified long-term outcomes in this study are survival rates, growth outcomes, neurodevelopmental outcomes, feeding behavior, as well as hearing and vision impairments. Growth and neurodevelopmental outcomes will be assessed at 0, 2, 4, 6, 9, 12, 15, 18 and 24 months of corrected age as well as at 3, 4, 5, 6, 7 and 8 years old.

**Discussion:**

The CIPTO study is the first prospective cohort in Indonesia focusing on preterm infants born at the CMGH. With a follow up until 8 years old, this study may provide useful insights to generate an evidence-based, Indonesia’s health care reference in managing premature infants and ensuring the optimum outcomes of ex-preterm infants.

**Supplementary Information:**

The online version contains supplementary material available at 10.1186/s12887-023-04263-z.

## Background

Indonesia is one of the most highly populated countries, with more than 5 million births annually. Preterm birth, defined as birth before 37 weeks of gestational age, occurs in 10–15% of Indonesians [1]. With approximately 600,000 preterm births every year, this would potentially generate social-economic impacts to the national healthcare [2–4]. In the recent years, Indonesia has improved its preterm care, shifting the paradigm from “helping babies to breath” to “helping babies to grow” [5]. It thus imposes substantial tasks for the healthcare services in Indonesia to manage those ex-preterm infants in the community settings and to ensure their quality of life.

Preterm birth cases in Indonesia mainly handled at government-owned academic hospitals. Cipto Mangunkusumo General Hospital (CMGH) is the top referral academic hospital in Indonesia, reporting more than 1,000 live births annually. Of note, more than half of those are preterm births, approximately 500–600 births [6]. It is also known that CMGH is one of the busiest neonatal intensive care unit (NICU) as well as one of the most advanced sites for treating preterm infant care in Indonesia. No long-term study on preterm infants in Indonesia has been conducted, therefore we aim to evaluate growth and development on ex-preterm infants until the pre-pubertal stage.

We therefore designed a prospective cohort study of preterm infants born at CMGH to observe long-term outcomes of preterm birth, particularly on the growth and developmental milestones. The Cohort of Indonesian Preterm Infants for Long-term Outcomes (CIPTO) study is the first and expected to be the largest prospective paediatric cohort in Indonesia. Aims of the CIPTO study are determining the outcomes of those ex-preterm infants and generating an evidence-based reference of preterm care to achieve optimum outcomes [7]. The CIPTO study will be a long-term study as it follows the ex-preterm infants until 8 years old (i.e., school-age children). This report describes its study protocol and pre-specified outcomes, hence allowing other neonatal centers to replicate this study.

## Methods

### Study design

**Fig. 1:**
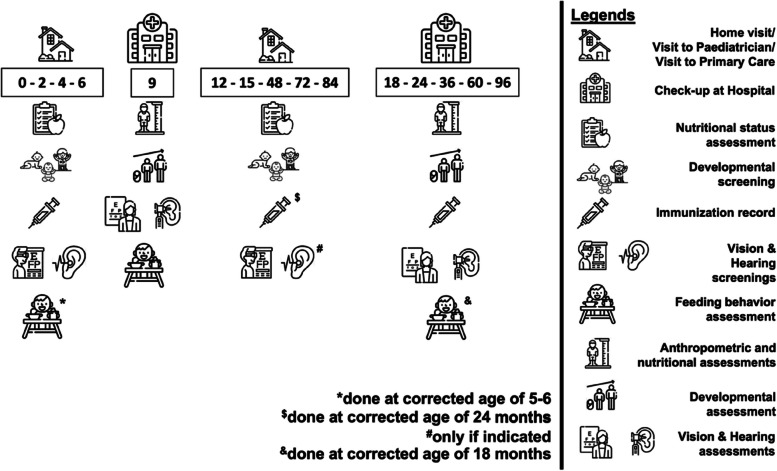
Post-discharge data collection of ex-preterm infants at various time points. The corrected age is used to determine time points of collecting data until 24 months. Subsequent assessments will be performed annually from 3 to 8 years old. Each number indicates the age in months. Interpretations of each icon are presented at the left side of this figure. All icons are provided by https://www.flaticon.com

This is a prospective cohort study conducted at the CMGH. All inborn infants are assessed for their eligibility and the participating infants will be followed from birth until discharge (as the baseline data). The sub-categories of preterm birth by the World Health Organization is adopted, i.e.: moderate to late preterm (32 to 37 weeks); very preterm (28 to less than 32 weeks); and extremely preterm (less than 28 weeks). In addition, the gestational age threshold to start treatment of preterm infants is minimum 26 weeks (i.e., a preterm birth less than 26 weeks is currently considered as a nonviable miscarriage). Furthermore, those survival preterm infants will be closely monitored until 8 years old. Outborn infants will be excluded because their data may be incomplete and they might receive inadequate treatments before being admitted into the CMGH [8, 9]. This study had been designed in 2019 and the data collection was started in 2020. As the COVID pandemic hit Indonesia since early 2020, the data collection was temporary withheld. Nevertheless, the data collection was re-started from early 2022. A detailed flowchart of the CIPTO study is presented in Supplementary Fig. 1, while types of post-discharge data collected from the participating subjects and the respective time points are depicted in Fig. 1.

### Setting

The CMGH is a government-owned public hospital, which also serves as the main academic hospital for the Faculty of Medicine at Universitas Indonesia. The CMGH is a tertiary center serves as the national reference hospital. It has a sophisticated NICU and greater coverage of diagnosis-related group reimbursement from the National Health Insurance, hence current international guidelines could be implemented with several limitations. Maternal and neonatal treatments would be administered to all patients, therefore, according to the recent guidelines without any economic barriers. The CMGH has the most advanced NICU in Indonesia, in which it is supported by a parenteral nutrition center, advanced ventilator devices (e.g., high-frequency oscillatory ventilator and neurally adjusted ventilatory assist), near-infrared spectroscopy, inhaled nitric oxide gas, amplitude-integrated electroencephalography, bedside ultrasound and echocardiography. Furthermore, the management of preterm infants at the CMGH, as an academic hospital, involves other paediatric specialists and other related departments as it is supported by the microbiological and pathological laboratories. The CMGH also able to performed complex surgery involving other departments. This collaboration facilitates routine discussion of cases to ensure that effective treatment shall be provided for each preterm infant.

### Participants

The inclusion criteria are infants discharged from the neonatal unit of CMGH with (i) born less than 37 weeks of gestational age without any restriction on birth weight; (ii) born at the CMGH; (iii) live in greater area of Jakarta; and (iv) written informed consent obtained from respective parents. The exclusion criteria are (i) multiple congenital anomalies; (ii) suspected of syndrome or chromosomal abnormalities; (iii) parents unwilling to participate; and (iv) self-discharge against medical advice. The drop-out criterion is patients relocate out of Jakarta during the first 24 months of the follow-up period, decline to participate during the study and unable to be contacted during the study period.

### Predictor variables

Potential predictors in this study are categorized into during hospital admission and after hospital discharge. Potential predictors obtained during hospital admission are gender; gestational age; anthropometric data at birth (weight, length and head circumference); maternal co-morbidities (preeclampsia [10], gestational diabetes [11, 12], intrauterine infection/chorioamnionitis [13], maternal infection and antepartum hemorrhage, as well as COVID-19); fetal distress [14]; history of resuscitation and its related information; antenatal steroid administration [15]; surfactant therapy; type and route of enteral and parenteral feedings; preterm-associated morbidities (sepsis [16], respiratory distress syndrome/hyaline membrane disease [17, 18], necrotizing enterocolitis [19, 20], patent ductus arteriosus [21], intraventricular hemorrhage [22, 23], other intracranial abnormalities, bronchopulmonary dysplasia [24, 25], invasive ventilation use and neonatal seizure [26]); history of blood product transfusion; anthropometric data at discharge (weight, length, head circumference, skinfold thickness and mid-upper arm circumference); parental height; sociodemographic background [27]; family history of prematurity; as well as hearing and vision impairment. Potential predictors after hospital discharge are type and route of feeding; immunization status; any occurrence of infectious disease (both as outpatient and inpatient), as well as growth and developmental assessments during routine visits. A detailed operational definition of each variable is presented in Supplementary Table 1.

### Outcomes

The pre-specified long-term outcomes in this study are survival rate; growth outcomes (stunted, wasting, undernutrition, obesity or microcephaly) [28, 29]; feeding behavior [30, 31]; neurodevelopmental outcomes [32, 33]; immunization record according to the current guideline of the Indonesian Paediatric Society [34]; hearing impairment [35]; and vision impairment, particularly retinopathy of prematurity [36]. Survival will be assessed in 6-month intervals by contacting the parents. Growth and neurodevelopmental outcomes will be assessed at 0, 2, 4, 6, 9, 12, 15, 18 and 24 months of corrected age and subsequently at 3, 4, 5, 6, 7 and 8 years old. The growth and development social paediatric specialist team and the nutrition and metabolic disease paediatric specialist team will assess the growth and development at 9, 18 and 24 months old of corrected age and at 3, 5 and 8 years old. The assessment is conducted by trained personnel (with the Developmental Pre-screening Questionnaire as well as Ages and Stages Questionnaire) and verified at the age of 9 months, 18 months and 3 years (with the Bayley Scales of Infant and Toddler Development 3rd edition) and at the age 5 years and 8 years (with the Griffith Scales of Child Development 3rd edition) by a paediatrician or trained psychologist. The Bahasa Indonesia version of those standardized tools are available and have been validated. At least at 9 months of corrected age, each participant will undergo laboratory examination for assessing deficiency of iron or other micronutrient. A referral to other specialist is possible whenever needed (e.g., neurology, respirology, hematology or endocrinology). Feeding behavior will be evaluated by using standardized caregiver-reported questionnaire at 5–6, 9 and 18 months of corrected age. The ear, nose and throat specialists will conduct hearing evaluations, based on the current screening guideline for neonates. A paediatric ophthalmologist will conduct vision evaluation, particularly for retinopathy of prematurity by using RetCam Envision^™^ or indirect ophthalmoscope. A detailed operational definition of each variable is presented in Supplementary Table 1. If an impairment is found at any time during the study period, the participant will be referred to receive an optimum care according to the updated local guideline.

### Data sources and collection

Inpatient data will be collected daily and prospectively. Anthropometric measurement is conducted by a trained nurse or physician using standardized tools. Information on parental background, maternal morbidities, preterm-related morbidities and neonatal care will be obtained from the medical records or direct observation. Diagnosis of diseases will be decided by attending physician based on the updated diagnosis criteria. The research team (including undergraduate medical students/paediatric residents/professional officers) will collect the data prior and after the hospital discharge.

After the discharge, patients should have routine monthly health visits during the first year, every 3 months during the second year and every year after 2 years according to guideline by the Indonesia’s Ministry of Health. In this study, we will focus to obtain data at 0, 2, 4, 6, 9, 12, 15, 18 and 24 months of corrected age and annually until 8 years of age. A delayed time of health visit will be rounded to the previous complete months (e.g., home visit at 3 months old is recorded as data at 2 months old). We will communicate with parents routinely to prevent any delay.

The health visit would be performed as scheduled home visit or as routine visit to primary care or paediatric clinic as well as mandatory visit to the CMGH. Data during home visits will be obtained by inspecting the children’s routine health check-up books (direct observation or photographs via mobile phone). As an alternative to home visit, parents could take their children to primary care or paediatric clinic for routine visits and subsequently report the results to the research team. In Jakarta, all primary care clinics have standardized and routinely calibrated anthropometric tools and measurement techniques, under supervision by the provincial and regional health bureaus. The follow-up data on 9, 18 and 24 months of corrected age and 3, 5, and 8 years old will be obtained from the mandatory visits to outpatient clinics at the CMGH. During those visits, the subject will have complete growth and developmental assessments by specialists to validate information obtained during routine home visit.

### Data management

Data will be stored in internal servers operated by the Center for Health Economics and Policy Studies (CHEPS) at the Faculty of Public Health, Universitas Indonesia. Access for data extraction is only provided to the investigators, in order to protect privacy of all subjects. Any further request for secondary data analysis must obtain permission from all investigators.

### Dissemination of results

Research results will be published in scientific journals and will be presented at scientific conferences as well. Of note, all paediatric clinical guidelines in Indonesia are regularly created and disseminated by the Indonesian Pediatric Society to all pediatricians in Indonesia. There are multiple working units within the Indonesian Pediatric Society, specializing in various health issues of pediatric population. With regard to the preterm infants, the Neonatology working unit is responsible to create relevant guidelines and disseminate them through regular conferences to practicing neonatologists. Results of this study will be shared with the Neonatology working unit to create a reference or guideline to manage surviving preterm infants in Indonesia.

### Potential bias

Potential selection bias is noticed due to a decision of not analysing patients who either self-discharged against medical advice or relocated out of Jakarta during the first 24 months of the follow-up period. In order to minimize that bias, close contact with parents will be maintained and the parents will be supported (through parent support group) to prevent self-discharge against medical advice or loss to follow-up. When it is unavoidable, the baseline characteristics between drop-out subjects and study participants will be compared to assess potential confounders or bias sources. The reason of dropping out could be neither related to the observation nor the outcome of interest as well. Thus theoretically would not introduce bias to the data analysis.

Information bias, specifically observation bias, could occur mainly during the anthropometric measurement, although this study will deploy trained personnel and standardized tools. A growth analysis by using percentiles or z-scores might be a way to minimize misclassification bias due to the measurement error. Moreover, paediatric evaluations at 9, 18 and 24 months of corrected age, as well as at 3, 5 and 8 years of age would confirm the previous anthropometric measurements and developmental evaluations conducted in the community setting.

### Study size

Several minimum sample size calculations were used to provide an adequate statistical power. For estimating the proportion of outcome (incidence) and 10% accuracy within the true value (e), the minimum sample size is 486 subjects, including potential 10% of drop-out and 15% of loss to follow-up. For estimating the survival, the minimum sample size is 193 subjects at the end of 8-year monitoring. For hypothesis testing of predictors with the mentioned study outcomes, the minimum sample size is 251 subjects, including potential 10% of drop-out and 15% of loss to follow-up. All sample size calculations were performed with a formula proposed by Lwanga and Lemeshow [37]. We have decided to recruit at least 500 subjects and the recruitment period requires approximately 2 years (i.e., before 2024).

### Statistical methods

Descriptive statistics on baseline characteristics of subjects will be visualized in a table or graph. Continuous data with normal distribution will be presented as mean and standard deviation, while the remaining will be presented as median, interquartile range, minimum and maximum. Categorical data will be presented as numbers of observation and percentage. Data distribution analysis using the Shapiro Wilk statistics test with p-value more than 0.05 indicates a normally distributed data. Bivariate analysis of continuous data using independent t-test or one-way ANOVA (parametric test) and Mann-Whitney U or Kolmogorov-Smirnov test (non-parametric test). Categorical data will be analyzed using a 2 × 2 table using the Chi-square test or Fischer’s exact test.

The main effect of interest is the relative risk (RR) and hazard ratio (HR) of pre-specified outcomes. Stratification analysis will be performed to evaluate possible confounder or effect modifier. A confounder is a variable related to predictors, causing the outcomes but not an intermediate variable. Several variables might be treated as predictors or confounders, depending on the analysis of causal relationships. The multivariate analysis will use generalized regression model with an adjusted RR as the main effect or Cox proportional hazard regression with an adjusted HR. Best causal or predictor model of each outcome analysis will be determined based on the most robust statistical model with satisfying discrimination and calibration performance, in which the chosen model will consider the basic disease mechanism. Data transformation or recoding will be conducted if a variable does not meet the criteria for the regression analysis.

Taken together, the CIPTO study is designed to improve long-term outcomes for preterm infants in Indonesia. A close monitoring and rigorous data analyses are expected to produce a new evidence-based reference for preterm care and to improve the overall quality of life of surviving infants in Indonesia.

### Supplementary Information

**Additional file 1: Supplementary Figure 1. **The flowchart of CIPTO study.** Supplementary Table 1. **The operational definition of CIPTO study.

## Data Availability

Not applicable.

## Abbreviations

AABR: Automated Auditory Brainstem Response
ASQ: Ages and Stages Questionnaire
BEBQ: Baby Eating Behavior Questionnaire
BPD: Bronchopulmonary dysplasia
CHEPS: Center for Health Economics and Policy Studies
CIPTO: Cohort of Indonesian Preterm Infants for Long-term Outcomes
CMGH: Cipto Mangunkusumo General Hospital
ENT: Ear Nose and Throat
EUGR: Extrauterine growth restriction
HR: Hazard ratio
IVH: Intraventricular haemorrhage
NEC: Necrotizing enterocolitis
NICU: Neonatal intensive care unit
OAE: Otoacoustic Emissions
PDA: Persistent ductus arteriosus
PPM: Person per month
RR: Relative risk
SD: Standard deviation
