# Supplementary material for: The Cohort of Indonesian Preterm Infants for Long-term Outcomes (CIPTO) study: a protocol
Source: BMC Pediatr. 2023 Oct 19;23:518. doi: 10.1186/s12887-023-04263-z (PMC10585843; doi:10.1186/s12887-023-04263-z)
Supplement: Supplementary file 1 — Additional file 1: Supplementary Figure 1. The flowchart of CIPTO study. Supplementary Table 1. The operational definition of CIPTO study. [file 12887_2023_4263_MOESM1_ESM.docx]

**Supplementary Figure 1.** The flowchart of CIPTO study.


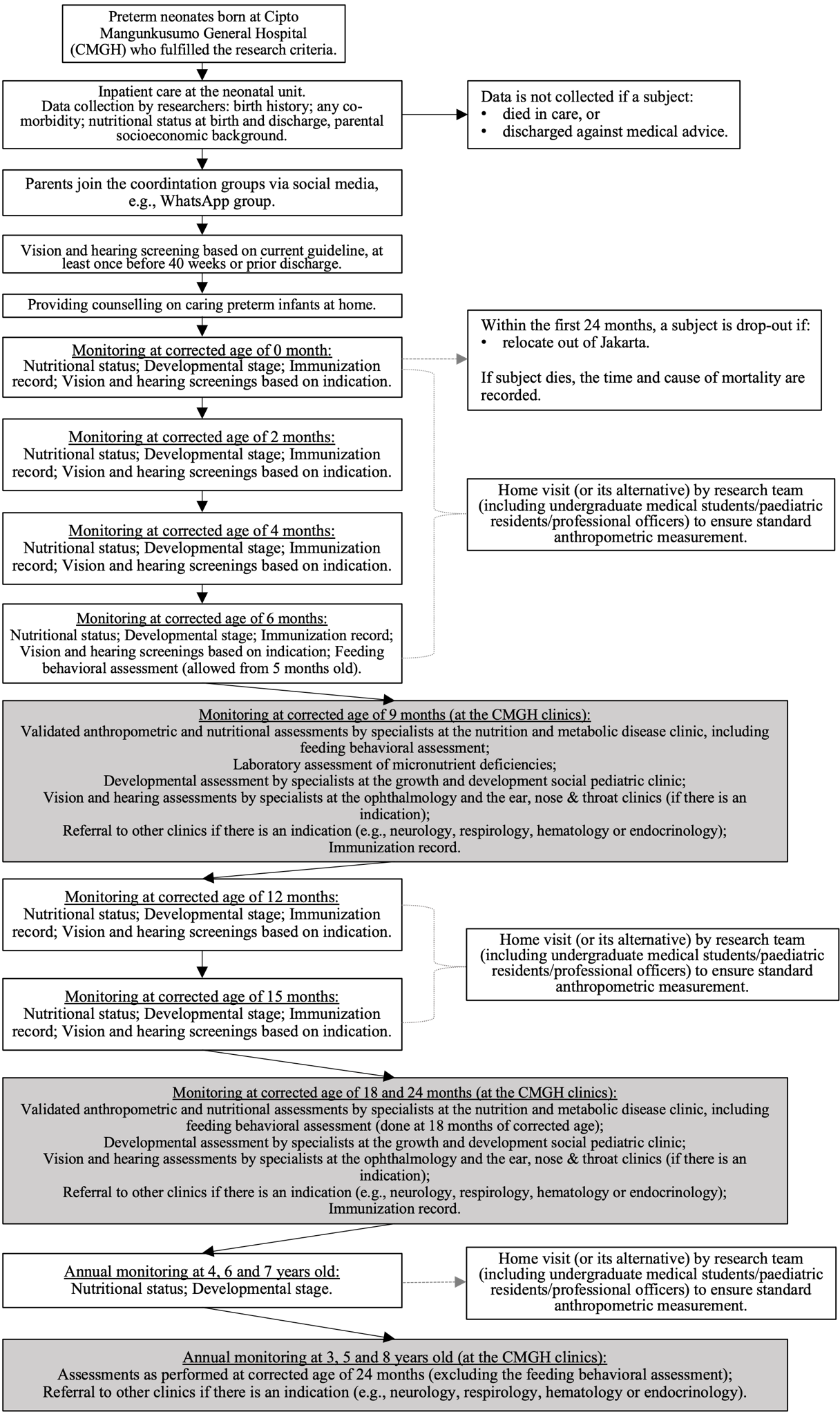


**Supplementary Table 1.** The operational definition of CIPTO study.

| **Variable** | **Definition** | **Measurement** | **Scale** | **Unit** | **Category** | **Measurement Time** |
| --- | --- | --- | --- | --- | --- | --- |
| Sex | Sex of neonate | Sex is determined by a physician based on the physical examination | Categorical |  | 0: Female 1: Male | Immediately after birth |
| Gestational age | Gestational age at birth | Fetomaternal ultrasound result confirmed by the Ballard score or last menstrual period time. Fetomaternal ultrasound is performed by an obstetrician-gynecologist. Ballard score is calculated by a paediatrician. | Numerical | week |  | Assessment was performed before delivery (ultrasound) or in the first 72 hours of life (Ballard) |
| Birth weight | Neonatal weight measured at birth | Weighing with a calibrated scale with a measurement accuracy of 1 gram. | Numerical | gram |  | At birth |
| Birth length | Neonatal length measured at birth | Measurement of body length in the supine position by using a measuring instrument with a measurement accuracy of 1 centimeter. | Numerical | centi-meter |  | At birth |
| Head circumference | Diameter of neonatal head circumference at birth | Measurement using a head circumference measuring tape with an accuracy of 1 centimeter. | Numerical | centi-meter |  | At birth |
| Anthropometric data at birth | Conformity of birth weight and gestational age | Birth weight and gestational age were plotted on the Lubchenco curve. | Categorical |  | 1:Appropriate for gestational age 2: Small for gestational age 3: Large for gestational age | At birth |
| APGAR score at 1 and 5 minutes after birth | Neonatal scoring with the APGAR scoring system | Assessment is carried out at 1 and 5 minutes after birth. The score comprises assessments of appearance, pulse, grimace, activity and respiratory. | Numerical | score |  | At 1 and 5 minutes after birth |
| Fetal distress [14] | Fetal disturbance marked by fetal heart rates beyond of normal limits (more than 160x/minute or less than 120x/minute) | Assessment by obstetrician-gynecologist. | Categorical |  | 0: No 1: Yes | Before delivery |
| Prenatal steroid administration [15] | Administration of steroid in pregnancy less than 34 weeks, with an aim to mature the lungs | Patient record. | Categorical |  | 0: No 1: Yes | Before delivery |
| Prenatal antibiotic administration | Administration of antibiotic before delivery | Patient record. | Categorical |  | 0: No 1: Yes | Before delivery |
| History of neonatal resuscitation | Providing respiratory support to neonate according to the neonatal resuscitation algorithm of the Indonesian Paediatric Society | Patient record. | Categorical |  | 0: No 1: Yes | At birth |
| Ventilation support during neonatal resuscitation | Type of ventilation support during neonatal resuscitation | Patient record.  Information from the physician who performed the resuscitation. | Categorical |  | 1:Without breathing aids 2: Oxygen Supplement-ation 3: Continuous Positive Airway Pressure (CPAP) 4: Nasal Intermittent Positive Pressure Ventilation (NIPPV) 5:Endo-  tracheal intubation | At birth |
| Maternal comorbidities | Disease or disorder experienced by pregnant women that affecting pregnancy outcomes | Patient record, preeclampsia, gestational diabetes, premature rupture of membranes, intrauterine infection, urinary tract infection, and chorioamnionitis. | Categorical |  | 0: No 1: Yes | Before delivery |
| Preeclampsia [10] | Pregnancy complication marked by an increase of blood pressure and proteinuria, with other potential symptoms. | Patient record. Diagnosis by an obstetrician-gynecologist. | Categorical |  | 0: No 1: Yes | Before delivery |
| Gestational diabetes [11, 12] | Diabetes occurs during pregnancy | Patient record. Diagnosis by an obstetrician-gynecologist. | Categorical |  | 0: No 1: Yes | Before delivery |
| Premature rupture of membranes | Rupture of membrane before delivery | Patient record. Diagnosis by an obstetrician-gynecologist. | Categorical |  | 0: No 1: Yes | Before delivery |
| Chorioamnionitis [13] | Infection on amniotic membrane, umbilical cord, or amniotic fluid | Patient record. Diagnosis by an obstetrician-gynecologist. | Categorical |  | 0: No 1: Yes | Before delivery |
| Urinary tract infection | Infection in the maternal urinary tract, with or without symptom | Patient record. Diagnosis by an obstetrician-gynecologist. | Categorical |  | 0: No 1: Yes | Before delivery |
| History of neonatal comorbidities | History of intraventricular hemorrhage (IVH), bronchopulmonary dysplasia (BPD), necrotizing enterocolitis (NEC), significant persistent ductus arteriosus (PDA), use of ventilator, blood transfusion, and nutrition intake during neonatal period | Patient record. | Categorical |  | 0: No 1: Yes | During treatment in the neonatal unit |
| Sepsis neonatorum | There are clinical signs of sepsis occurring at age of < 72 hours (early) or > 72 hours (late) and confirmed by results of blood or body fluid cultures, or clinically not improving after receiving antibiotic for the first 2 days | Patient record. Diagnosis by an attending physician. | Categorical |  | 0: No 1: Yes | During treatment in the neonatal unit |
| Necrotizing enterocolitis [19, 20] | There are clinical signs of intolerance to drinking, which is supported by a radiological imaging of pneumatosis intestinalis (degree > 2) using Bell’s criteria | Patient record. Diagnosis by an attending physician. | Categorical |  | 0: No 1: Yes | During treatment in the neonatal unit |
| Intraventricular hemorrhage [22, 23] | Observation of bleeding within the ventricles of the brain (grade > 2) through head imaging | Patient record. Diagnosis by an attending physician. | Categorical |  | 0: No 1: Yes | During treatment in the neonatal unit |
| Broncho-  pulmonary dysplasia [24, 25] | Infant with a history of receiving oxygen with a fraction of > 25% for 28 consecutive days | Patient record. Diagnosis by an attending physician. | Categorical |  | 0: No 1: Yes | During treatment in the neonatal unit |
| Persistent ductus arteriosus with hemodynamic disturbance [21] | Persistent ductus arteriosus is found with symptoms of hemodynamic disturbance via echocardiography | Patient record. Diagnosis by an attending physician. | Categorical |  | 0: No 1: Yes | During treatment in the neonatal unit |
| Mechanical ventilation | Infant receives a endotracheal tube intubation for more than 3 hours | Patient record. | Categorical |  | 0: No 1: Yes | During treatment in the neonatal unit |
| Severe respiratory distress syndrome [17, 18] | Infant has asphyxia at birth, which is supported by radiological examination showing a ground glass appearance | Patient record. Diagnosis by an attending physician. | Categorical |  | 0: No 1: Yes | During treatment in the neonatal unit |
| Retinopathy of prematurity | Retinal vasculature was observed during an examination with an indirect ophthalmoscope | Patient record. Diagnosis by a paediatric ophthalmic specialist. | Categorical |  | 0: No 1: Yes | During treatment in the neonatal unit |
| Nutritional Route | Nutritional route received by a premature infant during care in the neonatal unit | Patient observation in the neonatal unit. | Categorical |  | 1: enteral only 2: enteral and parenteral | During treatment in the neonatal unit |
| Highest parenteral protein concentration | The highest protein concentration obtained during parenteral nutrition | Patient observation in the neonatal unit. | Numerical | gram/kg/  day |  | During treatment in the neonatal unit |
| Highest parenteral lipid concentration | The highest lipid concentration obtained during parenteral nutrition | Patient observation in the neonatal unit. | Numerical | gram/kg/  day |  | During treatment in the neonatal unit |
| Type of enteral nutrition | Type of enteral nutrition given during treatment in the neonatal unit | Patient observation in the neonatal unit. | Categorical |  | 1: Breast milk  2: Donor breast milk  3: Standard formula  4: Premature formula  5: Special formula  6: Breast milk + Human milk fortifier | During treatment in the neonatal unit |
| Extrauterine growth restriction (EUGR) | Weight is less than the 10th percentile or deviating more than 2 standard deviations (SD) from birth weight | Matching weight to the chronological age on the Fenton curve. | Categorical |  | 0: No 1: Yes | At 36 weeks of gestational age or discharge from the neonatal unit |
| History of blood transfusion | Blood transfusion given during treatment in the neonatal unit | Patient observation in the neonatal unit. | Categorical |  | 0: No 1: Yes | During treatment in the neonatal unit |
| Duration of using an invasive mechanical ventilation | Duration of using an invasive breathing apparatus (ventilator) during treatment in the neonatal unit | Patient observation in the neonatal unit. | Numerical | day |  | During treatment in the neonatal unit |
| Hyper-  bilirubinemia | Pathological hyperbilirubinemia observed during treatment in the neonatal unit and when receiving an intervention | Patient observation in the neonatal unit. | Categorical |  | 0: No 1: Yes | During treatment in the neonatal unit |
| Use of ototoxic drugs | Administration of drugs that are known to be ototoxic | Patient observation in the neonatal unit. | Categorical |  | 0: No 1: Yes | During treatment in the neonatal unit |
| Family history of deafness | Congenital history of deafness in the family of subject | Interview. | Categorical |  | 0: No 1: Yes | During treatment in the neonatal unit |
| Intracranial radiological abnormalities | Results of intracranial examination during treatment in the neonatal unit | Examination using head ultrasound and/or other examinations as indicated (CT Scan, MRI) | Categorical |  | 0: No 1: Yes | During treatment in the neonatal unit |
| Results of the RetCam examination | Results of the RetCam examination performed by staffs of the paediatric ophthalmic division at the CMGH. | Patient records based on the first examination and follow-up examinations according to the recommendation. | Categorical |  | 0: normal 1: abnormal | During treatment in the neonatal unit and the subsequent visit as recommended by an ophthalmologist |
| Otoacoustic Emissions (OAE) result | OAE examination performed by staffs of the Ear Nose and Throat (ENT) department at the CMGH. | Patient records based on the first examination and follow-up examinations according to the recommendation. | Categorical |  | 0: pass 1: refer | During treatment in the neonatal unit and the subsequent visit as recommended by an ENT specialist |
| Automated Auditory Brainstem Response (AABR) result | AABR examination performed by staffs of the ENT department at the CMGH | Patient records based on the first examination and follow-up examinations according to the recommendation. | Categorical |  | 0: pass 1: refer | During treatment in the neonatal unit and the subsequent visit as recommended by an ENT specialist |
| Body weight | Subject's body weight measured after discharge | Weighing with a scale calibrated to an accuracy of 1 gram. The assessment is conducted by trained officers | Numerical | kilogram |  | At corrected age of 0, 2, 4, 6, 9, 12, 18 and 24 months as well as 3, 4, 5, 6, 7, and 8 years |
| Height | Subject's body length measured after discharge | Measurement of height with a measuring board (for subjects less than 24 months old) or microtoise (for subjects more than 24 months old) with an accuracy of 1 centimeter. The assessment is conducted by trained officers | Numerical | centi-meter |  | At corrected age of 0, 2, 4, 6, 9, 12, 18 and 24 months as well as 3, 4, 5, 6, 7, and 8 years |
| Head circumference | Subject's head circumference measured after discharge | Measurement of head circumference with a measuring tape by measuring the largest part of the subject's head. The assessment is conducted by trained officers | Numerical | centi-meter |  | At corrected age of 0, 2, 4, 6, 9, 12, 18 and 24 months as well as 3, 4, 5, 6, 7, and 8 years |
| Size of large fontanelle | Subject's large fontanelle measured after discharge | Length and width measurement of the large fontanelle prior to the closure. The assessment is conducted by trained officers | Numerical | centi-meter |  | At corrected age of 0, 2, 4, 6, 9, 12, 18 and 24 months as well as 3, 4, 5, 6, 7, and 8 years |
| Developmental status | Developmental status measured after discharge | Assessment of subject’s development with the Developmental Pre-screening Questionnaire and Ages and Stages Questionnaire (ASQ). The assessment is conducted by trained personnel and verified at the age of 9 months, 18 months and 3 years by a paediatrician or trained psychologist using standardized tools (Bayley III or other tools). Griffith Scales of Child Development 3rd edition or equal test is used for infants at the age of 5 and 8 years. | Categorical |  | 0: according to age 1: suspecting a delay | At corrected age of 0, 2, 4, 6, 9, 12, 18 and 24 months as well as 3, 4, 5, 6, 7, and 8 years |
| Stunting | Length/height less than -2 SD on the WHO height-for-age curve | Length/height measurement is plotted on the WHO curve. Assessment is performed by the research team and confirmed by a paediatric nutrition specialist at the corrected age of 9, 18 months, 3, 5, and 8 years old. | Categorical |  | 0: normal 1: stunting | At corrected age of 0, 2, 4, 6, 9, 12, 18 and 24 months as well as 3, 4, 5, 6, 7, and 8 years |
| Underweight | Weight less than -2 SD on the WHO weight-for-height curve | Weight measurement is plotted on the WHO curve based on the ideal body weight. Assessment is performed by the research team and confirmed by a paediatric nutrition specialist at the corrected age of 9, 18 months, 3, 5, and 8 years old. | Categorical |  | 0: normal 1: underweight | At corrected age of 0, 2, 4, 6, 9, 12, 18 and 24 months as well as 3, 4, 5, 6, 7, and 8 years |
| Wasting | Weight less than -3 SD on the WHO weight-for-height curve | Weight measurement is plotted on the WHO curve based on the ideal body weight. Assessment is performed by the research team and confirmed by a paediatric nutrition specialist at the corrected age of 9, 18 months, 3, 5, and 8 years old. | Categorical |  | 0: normal 1: wasting | At corrected age of 0, 2, 4, 6, 9, 12, 18 and 24 months as well as 3, 4, 5, 6, 7, and 8 years |
| Microcephaly | Head circumference less than -2 SD on the WHO weight-for-height curve | Head circumference is plotted on the Lubchenko curve. Assessment is performed by the research team and confirmed by a paediatric nutrition specialist at the corrected age of 9, 18 months, 3, 5, and 8 years old. | Categorical |  | 0: normal 1: micro-  cephaly | At corrected age of 0, 2, 4, 6, 9, 12, 18 and 24 months as well as 3, 4, 5, 6, 7, and 8 years |
| Developmental disorders | Developmental disorders in children who do not meet the developmental milestones.  Developmental assessments comprise of gross motor, fine motor, language, personal-social, and behavior | Extended assessment on subject which had risk of developmental delay after screening. Diagnosis of developmental delay or developmental disorder by growth and development social pediatric specialist. | Categorical |  | 0: normal 1: disorder | At corrected age of 0, 2, 4, 6, 9, 12, 18 and 24 months as well as 3, 4, 5, 6, 7, and 8 years |
| Survival | Survived subjects out of all observed subjects | Observation via interview or confirmation on the mortality data. Date and cause of death are recorded by the research team. | Categorical |  | 0: alive 1: death | During treatment in the neonatal unit and at corrected age of 0, 2, 4, 6, 9, 12, 18 and 24 months as well as 3, 4, 5, 6, 7, and 8 years |
| Enteral nutrition after discharge | Nutritional sources received by subject after discharged from the neonatal unit | Interview with caretaker or parent. | Categorical |  | 1: Breast milk  2: Donor breast milk  3: Standard formula  4: Premature formula  5: Special formula  6: Breast milk + Human milk fortifier 7: Comple-  mentary foods | During treatment in the neonatal unit and at corrected age of 0, 2, 4, 6, 9, 12, 18 and 24 months as well as 3, 4, 5, 6, 7, and 8 years |
| Occurrence of infectious disease | Any episode of occurred infectious disease; not exclusive to hospitalization. | Interview with caretaker or parent | Numerical | episode |  | During routine visits |
| Parental heights | Parental heights (of both father and mother) | Direct assessment with mikrotoice. Each assessment is performed twice, in which the average value is obtained. | Numerical | centi-meter |  | During home visit |
| Feeding behavior | Feeding behavior of subject is assessed by researcher | Assessment with the validated Baby Eating Behavior Questionnaire (BEBQ). An interview is conducted by a trained officer. | Categorical |  | 0: No issue 1: There is an issue | At corrected age of 5, 9 and 18 months |
| Medical history of being treated in the hospital | Medical history of being treated in the hospital with all causes, excluding trauma after discharged of the neonatal unit | Interview with caretaker or parent. | Numerical | times |  | During treatment in the neonatal unit and at corrected age of 0, 2, 4, 6, 9, 12, 18 and 24 months as well as 3, 4, 5, 6, 7, and 8 years |
| Parental economic background | Monthly income or expenditure of both parents | Interview with parent. | Numerical | rupiah |  | During recruitment or home visit |
|  |  |  | Categorical | Based on monthly income | 0: Above minimum regional salary  1: Below minimum regional salary | During recruitment or home visit |
|  |  |  | Categorical | Based on monthly expen-diture per person per month (PPM)  [37] | 0: < 354,000 IDR PPM (poor)  1: 354,000 – 532,000 IDR PPM (vulnerable)  2: 532,000 – 1,200,000 IDR PPM (aspiring middle class)  3: 1,200,000 – 6,000,000 IDR PPM (middle class)  4: >6,000,000 IDR PPM (upper class) | During recruitment or home visit |
| Parental occupation | Occupation of both parents | Interview with parent | Categorical |  | 1: Civil servant  2: Military personnel/ Police  3: Private sector  4: Self-employed/ Entrepreneur  5: Labourer  6: Other | During recruitment |
| Paternal education | The highest formal education level of father | Interview with caretaker or parent. | Categorical |  | 1: No formal schooling 2: Elementary school or equivalent 3: Junior high school or equivalent 4: Senior high school or equivalent 5: Bachelor’s degree or higher | During recruitment or home visit |
| Maternal education | The highest formal education level of mother | Interview with caretaker or parent. | Categorical |  | 1: No formal schooling 2: Elementary school or equivalent 3: Junior high school or equivalent 4: Senior high school or equivalent 5: Bachelor’s degree or higher | During recruitment or home visit |
| Maternal  COVID-19 history | History of COVID-19 confirmed by positive laboratory test prior delivery | Interview with mother | Categorical |  | 1: No history  2: Diagnosed COVID-19 at delivery  3: Recovered from COVID-19 prior delivery | During recruitment |
| Maternal nutritional status | Nutritional status according to body mass index or other similar measurement tools | Interview with mother | Categorical |  | 1: Normal  2: Under-nutrition 3: Overweight 4: Obesity | During recruitment or home visit |
| Paternal age | Father’s age during the pregnancy | Interview with caretaker or parent. | Numerical | years old |  | During recruitment or home visit |
| Maternal age | Mother’s age during pregnancy | Interview with caretaker or parent. | Numerical | years old |  | During recruitment or home visit |
| Caretaker | A person who taking care the subject on daily basis | Interview with caretaker or parent. | Categorical |  | 1: parent 2: relative 3: babysitter | During recruitment or home visit |
